# Rescue retrograde coronary venous ethanol ablation of ventricular tachycardia storm in a patient with Lamin A/C cardiomyopathy: a case report

**DOI:** 10.1093/ehjcr/ytae235

**Published:** 2024-05-09

**Authors:** Maarten A J De Smet, Rene Tavernier, Mattias Duytschaever, Sébastien Knecht, Jean-Benoît le Polain de Waroux

**Affiliations:** Department of Cardiology, AZ Sint-Jan Hospital, Ruddershove 10, 8000 Brugge, Belgium; Department of Cardiology, AZ Sint-Jan Hospital, Ruddershove 10, 8000 Brugge, Belgium; Department of Cardiology, AZ Sint-Jan Hospital, Ruddershove 10, 8000 Brugge, Belgium; Department of Cardiology, AZ Sint-Jan Hospital, Ruddershove 10, 8000 Brugge, Belgium; Department of Cardiology, AZ Sint-Jan Hospital, Ruddershove 10, 8000 Brugge, Belgium

**Keywords:** Electrical storm, Ventricular tachycardia, LMNA cardiomyopathy, Ablation, Ethanol, LV summit, Case report

## Abstract

**Background:**

Left ventricular (LV) summit arrhythmias account for up to 14% of LV arrhythmias. The ablation of LV summit arrhythmias is challenging, as testified by the fact that radiofrequency (RF) catheter ablation failure is frequent. Retrograde coronary venous ethanol infusion has been proposed as an alternative approach for the ablation of LV summit arrhythmias.

**Case summary:**

A 47-year-old man with Lamin A/C cardiomyopathy was referred for the ablation of a pleiomorphic ventricular tachycardia (VT) storm, with dominant morphology compatible with LV summit origin. He first received a combined endo- and epicardial RF ablation with the elimination of three clinically relevant VTs. However, the dominant VT could not be ablated due to the proximity of the coronary vasculature and phrenic nerve and remained inducible. Accordingly, an urgent rescue redo procedure consisting of retrograde coronary venous ethanol ablation was performed. Based on the best pace-match and precocity, the first septal, retro-pulmonary branch and the first diagonal branch were infused with ethanol with immediate cessation of the tachycardia and non-inducibility. Anti-arrhythmic drugs were withdrawn, while guideline-directed medical therapy for heart failure was continued. No complications occurred. After 3 months, the patient remained free from any arrythmias.

**Discussion:**

Ablation of LV summit arrythmias is challenging, especially in the context of an electrical storm or in patients with structural heart disease. In such a situation, rescue ablation with retrograde coronary venous ethanol infusion represents an attractive alternative ablation modality.

**Learning points**

- The arrhythmogenic substrate in patients with LMNA cardiomyopathy includes scarring around the mitral annulus and deep intramural substrate with ventricular arrhythmias that may arise from the left ventricular (LV) summit.
- The arrhythmogenic substrate at the LV summit may be inaccessible for endo- and epicardial radiofrequency ablation due to the presence of the intramural substrate and due to the proximity of the coronary vasculature and/or phrenic nerve.
- Retrograde coronary venous ethanol infusion may be adopted as a rescue procedure to successfully treat a ventricular tachycardia storm arising from the LV summit.

## Introduction

Approximately 14% of left ventricular (LV) arrhythmias originate from the LV summit and may be idiopathic or associated with structural heart disease.^1^ The summit of the LV is the most superior portion of the epicardial LV and is bounded by an arc from the left anterior descending artery, superior to the first septal perforating branch, to the left circumflex coronary artery. The great cardiac vein bisects the LV summit in an inferior part, accessible for ablation, and a superior inaccessible part. As such, endo- and epicardial radiofrequency (RF) ablations of LV summit arrhythmias are associated with frequent ablation failure and ventricular arrhythmia (VA) recurrence.^1,2^ The LV venous circulation allows vascular access to the arrhythmia substrate in both idiopathic LV summit arrhythmias and structural heart disease. In these patients, retrograde coronary venous ethanol infusion has been proposed as an alternative ablation strategy.^3,4^

In this study, we report a case of successful alcohol ablation of an electrical storm with dominant ventricular tachycardia (VT) arising from the LV summit in a patient with Lamin A/C (LMNA) cardiomyopathy.

## Summary figure

**Table:** 

29–30 May 2023	Presentation with a haemodynamically unstable ventricular tachycardia (VT) storm, refractory to intravenous amiodarone. Partial control of ventricular arrhythmias following deep sedation. Diagnosis of non-ischaemic dilated cardiomyopathy, coronary angiography showed normal results
8 June 2023	Cardiac magnetic resonance imaging: left ventricular ejection fraction 24%, extensive late gadolinium enhancement intramurally at the basal septum, anterior wall, and lateral wall of the left ventricle
15 June 2023	Genetic analysis: a heterozygous missense variant in Exon 4 of the LMNA gene (c646C > T; p.Arg216Cys). Class 4 likely pathogenic variant
16 June 2023	Implantation of a dual-chamber implantable cardioverter defibrillator (ICD), hospital discharge on optimal medical therapy [beta-blocker, sodium–glucose cotransporter 2 (SGLT2) inhibitor, mineralocorticoid receptor antagonist (MRA), angiotensin receptor/neprilysin inhibitor (ARNI), and amiodarone]
5 July 2023	Start of an ambulatory cardiac rehabilitation programme
12 July 2023	Recurrence of the electrical storm with recurrent appropriate ICD interventions, refractory to deep sedation, beta-blocker, intravenous amiodarone, and xylocaine. The heart team recommends VT ablation
13 July 2023	Pre-operative cardiac computed tomography scan done
13 July 2023	Combined radiofrequency endo- and epicardial VT ablation. Successful ablation of four clinical VTs. Ablation of the LV summit VT limited by the proximity of the phrenic nerve and coronary arteries. LV summit VT remains inducible
18 July 2023	Rescue multi-vein retrograde coronary venous ethanol ablation. Non-inducible
20 July 2023	Successful weaning, cessation of anti-arrhythmic drugs, and intensive care unit discharge
24 July 2023	Hospital discharge on guideline-directed medical therapy for heart failure (beta-blocker, ARNI, SGLT2 inhibitor, MRA, diuretics, and no amiodarone)
25 August 2023	Successful recovery. Implantable cardioverter defibrillator interrogation: no arrhythmia episodes (VT monitor zone from 130 b.p.m.)
10 October and 26 October 2023	No problems reported at follow-up consultations. An uncomplicated cardiac rehabilitation programme initiated. Left ventricular ejection fraction 40% on echocardiography. Implantable cardioverter defibrillator interrogation: 1 episode of non-sustained VT (12 beats, 155 b.p.m.)

## Case presentation

**Figure 1:**
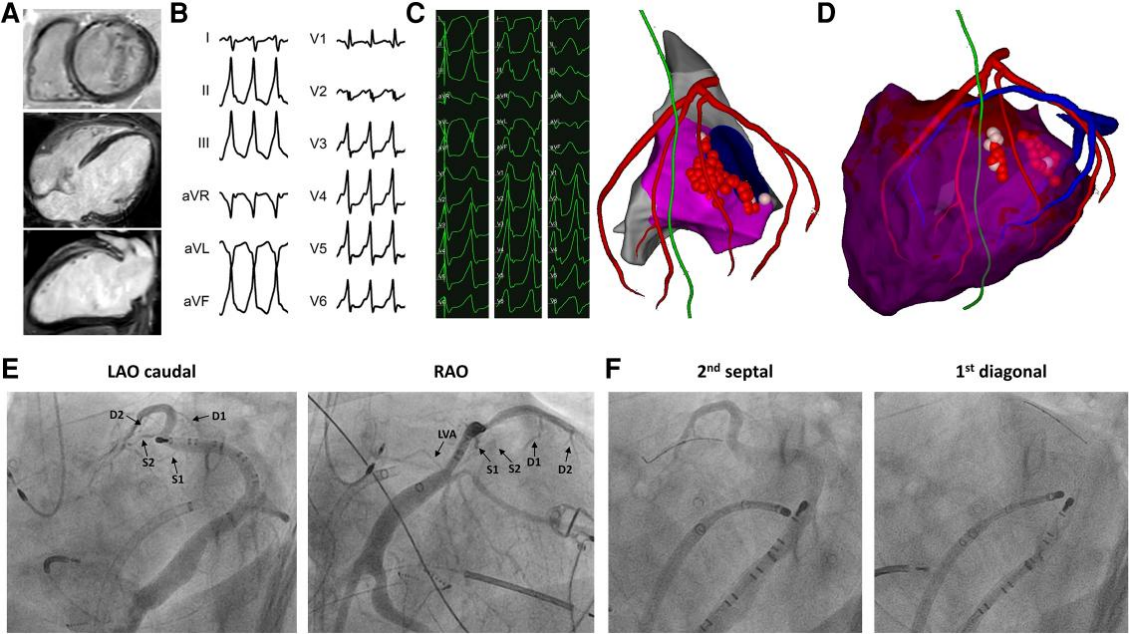
(*A*) Cardiac magnetic resonance images showing deep intramural late gadolinium enhancement around the mitral annulus. (*B*) A 12-lead echocardiography showing ventricular tachycardia with an inferior axis, rS pattern in V1-2, and sudden QRS complex transition in V3. A slow upstroke of the QRS complex suggests epicardial origin. Collectively, this indicates left ventricular summit tachycardia. (*C*) Anatomical and bipolar endocardial maps fused with segmented cardiac computed tomography images showing ablation sites with the successful elimination of three ventricular tachycardias. (*D*) Anatomical and bipolar epicardial maps fused with segmented cardiac computed tomography images showing a limited ablation set due to the inter-position of the phrenic nerve and coronary artery. (*E*) Left anterior oblique caudal and right anterior oblique images of coronary sinus angiography. LVA, left ventricular annular branch; S1, first septal branch; S2, second septal branch; D1, first diagonal branch; D2, second diagonal branch. (*F*) The left fluoroscopy image shows a selective distal cannulation of the second septal branch with a unipolar pacing wire and over-the-wire balloon. Here, the pace-match was 97% similar to the clinical tachycardia with a precocity of −52 ms to the onset of the QRS complex. The right fluoroscopy image shows a selective cannulation of the first diagonal branch with the unipolar pacing wire and over-the-wire balloon, showing similar excellent pace-match and precocity parameters.

We report the case of a 47-year-old patient without previous medical history who presented with a haemodynamically unstable VT storm, resistant to intravenous amiodarone and partially controlled with deep sedation. No reversible causes were present. The results of coronary angiography were normal. Echocardiography (ECG) and cardiac magnetic resonance (CMR) imaging showed a dilated cardiomyopathy with an LV ejection fraction (LVEF) of 24% and a deep intramural late gadolinium enhancement around the mitral annulus (*Figure 1A*). A genetic analysis revealed a Class 4 likely pathogenic variant in the LMNA gene (c646C > T; p.Arg216Cys, missense variant). Following weaning, the patient was treated with beta-blocker, mineralocorticoid receptor antagonist, sodium–glucose cotransporter 2 inhibitor, angiotensin receptor/neprilysin inhibitor, and amiodarone. He was implanted with a dual-chamber implantable cardioverter defibrillator (ICD) and was included in an ambulatory cardiac rehabilitation programme.

One month later, the patient presented with a drug-resistant (beta-blocker, amiodarone, and xylocaine) and sedation refractory pleiomorphic VT storm (278 ICD therapy in 1 day). Treatment options were discussed among the members of the heart team (electrophysiologists, heart failure specialists, intensivists, etc.) with options for VT ablation, stellate ganglion block, LV assist device, or urgent cardiac transplantation. The patient was referred for urgent VT ablation. The dominant VT with a cycle length of 460 ms showed an ECG morphology with an inferior axis, a sudden R/S transition in V3, and a maximum deflection index of 0.8, indicating epicardial origin. The findings are compatible with those of an LV summit VT (*Figure 1B*). He first received a combined endo- and epicardial RF ablation (*Figure 1C* and *D*). During this procedure under general anaesthesia, endo- and epicardial substrate maps of local activation time were prepared and bipolar voltage was performed using a high-density mapping catheter (Biosense Octaray). Following a coronary angiogram and guided by pre-operative computed tomography (CT) scan imaging, ablation at the LV summit was performed using a Biosense SmartTouch catheter. Epicardial ablation was limited because of the proximity of the left anterior descending artery and phrenic nerve (*Figure 1D*). At the end of this procedure, and despite the elimination of three clinically relevant VTs, the dominant VT remained inducible. Accordingly, an urgent rescue redo procedure consisting of retrograde coronary venous ethanol ablation was planned. The procedure was performed under general anaesthesia. A decapolar catheter was placed in the distal coronary sinus (CS) at the great cardiac vein/anterior interventricular vein junction. An ablation catheter was positioned in the LV outflow track at the best endocardial pace-match (86%) site. The distal CS venous anatomy was analysed using the venous phase of the CT scan (not shown) and confirmed using a venogram performed in left anterior oblique caudal (LAO-caudal) and right anterior oblique (RAO) views (*Figure 1E*). A coronary venogram was performed by advancing a sheath in the CS via the right femoral vein (Biosense Preface). Venograms were recorded in RAO and LAO-caudal views following the administration of a contrast injection over a 6 Fr internal mammary artery (IMA) guiding catheter using a syringe. Using a 2 Fr octopolar mapping catheter (Baylis EPstar 2F) or a 0.014″ angioplasty wire (Balance Middle Weight) and an 1.5–2.5 × 6–8 mm over-the-wire balloon through the guiding catheter, the different CS branches of interest were selectively cannulated and investigated for early potentials and pace-matching. The latter was achieved by connecting the angioplasty wire to an alligator clip in a unipolar configuration with a needle inserted in the skin as a reference electrode. The unipolar signal was looped to the CARTO system by custom-defining the wire using the catheter definition tool and by connecting to the patient interface unit. The over-the-wire balloon provides an isolation of the distal wire tip as a pacing and recording electrode. Before ethanol infusion (3–9 mL per CS branch), a selective venogram was performed following balloon inflation.

**Figure 2:**
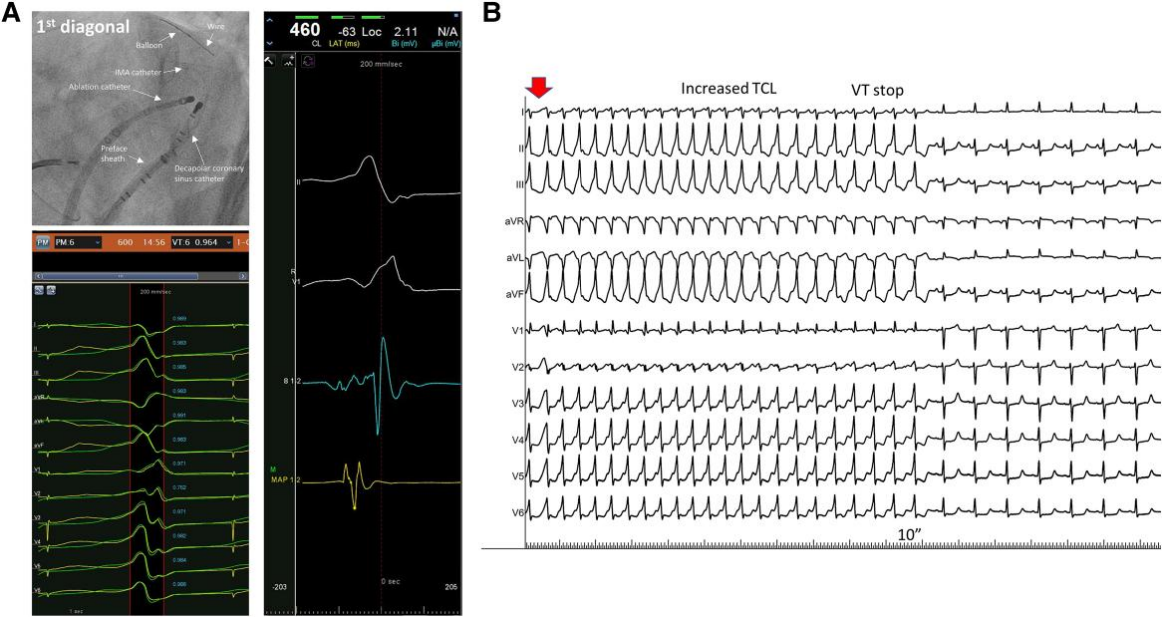
(*A*) Left upper panel: A left anterior oblique caudal view showing a decapolar coronary sinus catheter, an ablation catheter at the best endocardial pace-match region and a preface sheath with an internal mammary artery guiding catheter, and a selective cannulation of the first diagonal branch with an angioplasty wire and over-the-wire balloon. Left lower panel: a pace-match in the first diagonal branch. Right panel: an early presystolic signal (green trace) in the first diagonal branch during ventricular tachycardia. (*B*) Left ventricular summit ventricular tachycardia cycle lengths increase with the termination of ventricular tachycardia during ethanolization of the second septal branch.

First, the LV annular vein demonstrated no premature electrogram and a low pace-match with the clinical VT. However, mapping of the distality of the first septal branch revealed an early intracardiac electrogram signal (−52 ms from the beginning of the QRS complex), a unipolar QS pattern, and a good pace-match with the clinical VT (97%). Following a proximal selective occlusion of the first septal branch with the balloon, 6 mL of ethanol were injected. However, a slightly different VT (also from the LV summit) remained inducible. Further mapping into the distal second septal (retro-pulmonary branch) during VT demonstrated a very good precocity and unipolar characteristics (*Figure 1F*). An injection of 4 mL ethanol progressively prolonged the tachycardia cycle length until termination (*Figure 2B*). Finally, a last VT (arising from the peri-mitral region) was induced with a more aggressive induction protocol. The first diagonal branch was mapped with good precocity, a unipolar QS pattern, and a pace-match of 96% (*Figures 1F* and *2A*). Ethanol ablation (6 mL) was performed following selective balloon occlusion. Afterwards, the patient was non-inducible. No voltage map post-ethanol ablation was performed. The anti-arrhythmic drugs were withdrawn, while guideline-directed medical therapy for heart failure was continued. No complications occurred. After 3 months, the patient remained free from sustained arrythmias. The LVEF dramatically improved (LVEF 40%).

## Discussion

The current European Society of Cardiology guidelines for the treatment of cardiomyopathies and VT indicate a Class I indication for catheter ablation in the treatment of sedation- and drug-resistant electrical storm.^5,6^ Additionally, long-term management of VT, especially those that are monomorphic and scar-related, also requires an ablation of the arrhythmogenic substrate.^5^ In patients with cardiomyopathies, including LMNA cardiomyopathy, intramural and epicardial involvement of VT circuits are common, and the efficacy of ablation may depend on a combined endo- and epicardial RF catheter ablation or the use of rescue strategies, such as bipolar and needle ablation, ethanol ablation, radiotherapy, or surgical ablation.^5^ Pre-operative ECG and imaging, including cardiac CT and CMR, are crucial in characterizing the arrhythmogenic substrate and guiding an optimal treatment strategy.

Here, we report on a patient with LMNA cardiomyopathy and electrical storm arising from the LV summit that was successfully treated with retrograde coronary venous ethanol infusion as a rescue ablation strategy. In patients with LMNA cardiomyopathy, VT is often associated with basal scarring around the mitral annulus and an intramural substrate that may extend in the LV summit.^2^ Unfortunately, conventional endo- and epicardial RF catheter ablation for LV summit arrhythmias is associated with frequent ablation failure and VA recurrence.^1,2^ Both in patients with refractory LV summit arrhythmias and in patients with structural heart disease, retrograde coronary venous ethanol infusion has been proposed as an alternative strategy to ablate LV summit arrhythmias.^3,4^ Previous studies have reported on the feasibility (98% success rate, 23–42% recurrences) and safety (5% complications: cardiac tamponade or pericarditis) of retrograde coronary venous ethanol ablation of refractory VAs arising from the LV summit and of substrate ablation in the setting of structural heart disease.^3,4,7^ This report adds that ethanolization may provide effective substrate ablation in patients with an inaccessible substrate or a deep intramural scar in the setting of LMNA cardiomyopathy and electrical storm.

The feasibility and safety of coronary venous ethanol ablation is reported to be higher than that of coronary arterial ethanol infusion. For the latter, the procedural success rate is 56–84% with 33–64% recurrences. Adverse events are not uncommon following intracoronary ethanol infusion and include coronary artery dissection, thrombosis, myocardial infarction, pericarditis (10% of patients), and conduction block (40% of patients). Finally, variability in the coronary anatomy and the presence of coronary stenosis make coronary arterial ethanol infusion challenging and outcomes difficult to predict.^7^

## Conclusion

This case report is the first to propose retrograde coronary venous ethanol infusion as a safe and effective rescue strategy for electrical storm in a patient with LMNA cardiomyopathy. Early adoption of this technique in the case of an LV summit VT storm may be considered.

## Data Availability

The data underlying this article will be shared on reasonable request to the corresponding author.

## Lead author biography

Maarten A.J. De Smet is an electrophysiology and pacing fellow at the Department of Cardiology, AZ Sint-Jan Hospital, Brugge, Belgium.

**Consent:** The authors confirm that written consent for the submission and publication of this case report, including images and associated text, has been obtained from the patient in line with COPE guidance.

**Funding:** None declared.
